# Genomic insights into the adaptation of *Synechococcus* to the coastal environment on Xiamen

**DOI:** 10.3389/fmicb.2023.1292150

**Published:** 2023-11-20

**Authors:** Ting Zhang, Kun Zhou, Yanhui Wang, Jinxin Xu, Qiang Zheng, Tingwei Luo, Nianzhi Jiao

**Affiliations:** ^1^Fujian Key Laboratory of Marine Carbon Sequestration, Carbon Neutral Innovation Research Center, Xiamen University, Xiamen, Fujian, China; ^2^Department of Bacteriology, University of Wisconsin–Madison, Madison, WI, United States

**Keywords:** Xiamen coast, *Synechococcus*, metagenomics, mini-metagenomics, cyanophages

## Abstract

*Synechococcus* are widely distributed in the global ocean, from the pelagic zone to coastal waters. However, little is known about *Synechococcus* in coastal seawater due to limitations in isolation and culture conditions. In this study, a combination of metagenomic sequencing technology, flow cytometry sorting, and multiple displacement amplification was used to investigate *Synechococcus* in the coastal seawater of Xiamen Island. The results revealed 18 clades of *Synechococcus* and diverse metabolic genes that appear to contribute to their adaptation to the coastal environment. Intriguingly, some metabolic genes related to the metabolism of carbohydrates, energy, nucleotides, and amino acids were found in 89 prophage regions that were detected in 16,258 *Synechococcus* sequences. The detected metabolic genes can enable prophages to contribute to the adaptation of *Synechococcus* hosts to the coastal marine environment. The detection of prophages also suggests that the cyanophages have infected *Synechococcus*. On the other hand, the identification of 773 genes associated with antiviral defense systems indicates that *Synechococcus* in Xiamen have evolved genetic traits in response to cyanophage infection. Studying the community diversity and functional genes of *Synechococcus* provides insights into their role in environmental adaptation and marine ecosystems.

## Introduction

*Synechococcus* are a type of cyanobacteria found throughout the global oceans, from polar to temperate to tropical waters. They are estimated to be most abundant in nutrient-rich tropical and subtropical surface waters, such as the Indian Ocean and western Pacific, where their abundance can reach 3.4 × 10^4^ and 4.0 × 10^4^ cells mL^−1^, respectively (Flombaum et al., 2013). In contrast, their abundance in the Arctic and Southern Oceans is lower, at ~10^3^ cells mL^−1^ (Flombaum et al., 2013). *Synechococcus* are most abundant in shallow waters at a depth of 50 m (Fu et al., 2007), and they occupy a wide range of ecological niches and exhibit significant diversity in habitat, physiology, morphology, and metabolic capabilities. Based on phylogenetic relationship using 16S rRNA genes, marine *Synechococcus* have been classified into three major subgroups, designated 5.1, 5.2, and 5.3 (Scanlan et al., 2009).

*Synechococcus* play an important role in the regulation of global biogeochemical cycles and carbon fixation (Zehr, 2011; Cabello-Yeves et al., 2017). They contribute approximately 21% of the ocean primary productivity and are expected to continue to increase, with a projected increase of 14% in the tropics over the next 100 years (Flombaum et al., 2013). *Synechococcus* also serve as a food source for various eukaryotes, such as ciliates and flagellates (Coutinho et al., 2016), which are important in driving the circulation of matter and energy flow in the environment and maintaining the stability of Earth’s ecosystem (Falkowski et al., 2008). *Synechococcus* exhibit tremendous metabolic diversity, and previous studies have shown that these organisms have evolved unique strategies to adapt to their environment. These strategies include aspects of their metabolism and physiology, such as nutrient uptake and utilization, regulatory systems, and motility.

Cyanophages are known to have a significant impact on the population dynamics and evolution of *Synechococcus*. *Synechococcus* are frequently exposed to phage infections, which affect their population by killing a fraction of them daily (~0.005–30% daily) (Fedida and Lindell, 2017). Lysogeny of temperate cyanophages occurs in marine *Synechococcus* populations, for example, those situated in Tampa Bay, Florida (McDaniel et al., 2002). This suggests that *Synechococcus* in coastal areas have been infected by temperate cyanophages and harbor prophages in their chromosomes. Temperate phages can increase the size of the *Synechococcus* genome and contribute to host metabolism. As a result, cyanophages are expected to have a significant impact on the function and evolution of their cyanobacterial hosts. On the other hand, there is evidence indicating the widespread presence of the clustered regularly interspaced short palindromic repeat (CRIPSR)–CRISPR-associated protein (Cas) system in cyanobacteria (Cai et al., 2013), which suggests that *Synechococcus* have developed anti-cyanophage defense systems to protect against viral infection. Furthermore, the discovery of additional defense systems, such as the restriction-modification system (RM) (Ershova et al., 2015), the defense island system associated with restriction-modification (DISARM) of innate immunity (Ofir et al., 2018), and the bacteriophage exclusion system (BREX) (Goldfarb et al., 2015), provides further opportunities to explore potential defense mechanisms in *Synechococcus.*

The Xiamen Coastal Sea is located in the subtropical region bounded by the Taiwan Strait in the southeast and the Jiulong River in the southwest, which allows suspended matter and nutrients from nearby waters to enter the Xiamen Sea (Yu et al., 2015; Wang et al., 2019). The hydrological environment of the area is highly dynamic and influenced by various factors, such as freshwater from the Jiulong River, seawater from the South China Sea, and human activities. These factors may affect the diversity of *Synechococcus* communities in the area, and contribute to their distinct genetic characteristics. Although coastal *Synechococcus* bacteria have not been extensively studied due to limited isolation and culture conditions, analysis of their genomic information is crucial to understanding their roles in marine environments. The aims of this study were to sequence the metagenomes of *Synechococcus* clades from three stations (S03, S07, and S12) located in the coastal areas of Xiamen to improve our understanding of their composition and phylogeny, and to explore the molecular mechanisms of environmental adaptation, including metabolism and defense against viral infections.

## Materials and methods

### Sample collection

Seawater from each station (S03, S07, and S12) was initially prefiltered through a 20 μm mesh. Subsequently, 2 liters of the prefiltered seawater was further filtered using a 0.22 μm polycarbonate membrane (47 mm, Millipore, United States). The filtered polycarbonate membranes were preserved and immediately stored at −80°C for subsequent community structure analysis and metagenome sequencing. Concurrently, 2–4 mL of the prefiltered seawater was transferred to a sterile cryotube, and glycerol was added as a cryoprotectant to achieve a final concentration of 10% v/v. The samples were quickly frozen in liquid nitrogen and stored at −80°C for cell sorting.

### Total DNA extraction and metagenome sequencing

Total DNA of microorganisms on the filtered polycarbonate membranes of three stations (designated M8-S03, M8-S07, and M8-S12) was extracted using the HiPure Soil DNA 96 Kit (Magen, Guangzhou, China). All procedures were performed in accordance with the manufacturer’s guidelines. A library with an insert size of approximately 350 bp was constructed and sequenced on the Illumina HiSeq platform to generate 2 × 150 bp paired-end (PE) reads (Illumina, San Diego, CA, United States).

### Community structure analysis

To assess the community composition of microorganisms at each station, the 16S-23S rRNA internal transcribed spacers (ITSs) of *Synechococcus* genomes were amplified and sequenced. The 16S-23S rRNA ITS region was amplified through PCR using the 16S primer (5’-TGGATCACCTCCTAACAGGG-3′) and the 23S primer (5’-CCTTCATCGCCTcTGTGCC-3′) as previously detailed (Cai et al., 2010). The PCR was conducted in a final volume of 25 μL, which included 2.5 μL of TransStart Buffer, 2 μL of dNTPs, 1 μL of each primer, 0.5 μL of TransStart Taq DNA polymerase, and 20 ng of template DNA. The thermal profile for amplification was as follows: initial denaturation at 94°C for 5 min, then 25 cycles of denaturation at 94°C for 0.5 min, annealing at 56°C for 0.5 min, and extension at 72°C for 0.5 min, with a final extension at 72°C for 5 min. Subsequently, the amplicons were subjected to electrophoresis on a 1.5% agarose gel, and their concentration was determined using a multifunctional microplate reader (Tecan, Infinite M200 Pro, Switzerland). Then, the PCR products were sequenced using the PacBio third-generation sequencing platform.

### Cell sorting and mini-metagenomic sequencing of *Synechococcus*

Mini-metagenomics, which integrates the advantages of both shotgun and single-cell metagenomic analyses (Yu et al., 2017), was used to capture additional genomic signals from *Synechococcus*. We sorted 1,000 *Synechococcus* cells from each sample collected at three stations (S03: 3-S03, 6-S03, 8-S03, 12-S03; S07: 3-S07, 6-S07, 8-S07, 12-S07; S12: 3-S12, 6-S12, 8-S12, 12-S12) using a FACSAria flow cytometer (BD Biosciences). The cytometer was equipped with a solid-state laser providing 13 mW at 488 nm and was set to purity mode with a standard filter set-up. We extracted and amplified the genomic DNA of *Synechococcus* using the Discover-sc™ Single Cell Kit (Vazyme, China). All procedures were executed following the manufacturer’s guidelines. To ensure sterility, we placed the kit and consumables on a sterile operating table sterilized with 75% alcohol and ultraviolet light for 1 h before sorting. We assessed the quality and concentration of the DNA libraries using a QSEP100 bioanalyzer and Qubit 3.0, respectively. Then, we mixed the DNA libraries and subjected them to 2 × 150 bp PE sequencing using an Illumina HiSeq (Illumina, San Diego, CA, United States) instrument according to the manufacturer’s instructions.

### Metagenome assembly, binning, and annotation

The raw reads were trimmed by Trimmomatic version 0.36 with custom parameters (ILLUMINACLIP: TruSeq3-PE.fa:2:30:10 LEADING:3 TRAILING:3 SLIDINGWINDOW:4:15 MINLEN:40) (Bolger et al., 2014). Then, trimmed reads of metagenomes of microorganisms without sorting were assembled using metaSPAdes version 3.11.1 with default parameters (Bankevich et al., 2012). The trimmed reads of metagenomes of sorted microorganisms were assembled using SPAdes version 3.11.1 with custom parameters (−-sc, −-careful) (Bankevich et al., 2012). Each assembled metagenome was individually imported into MetaWRAP with custom settings: --metabat2, −-maxbin2, −-concoct, bin_refining. The assessment of metagenome-assembled genomes (MAGs) for contamination and completeness was conducted using CheckM (Parks et al., 2015). High-quality MAGs (≥50% completeness, ≤10% contamination, sequences ≥1 kb) were retained for subsequent analysis. The high-quality MAGs were taxonomically annotated with the BAT against the NCBI-nr database^1^ using default parameters (von Meijenfeldt et al., 2019), retaining genome bins of *Synechococcus*. Five *Synechococcus* genome bins were input into Anvi’o5^2^ to perform the average nucleotide identity analysis for genomic comparison with custom parameters (anvi-script-FASTA-to-contigs-db, anvi-run-ncbi-cogs, anvi-gen-genomes-storage, anvi-pan-genome, anvi-compute-ani, anvi-display-pan). Taxonomic classification of *Synechococcus* genome bins was based on single-copy genes identified by GTDB-Tk v0.3.1 with the parameter classify_wf (Parks et al., 2018). In addition, unbinned metagenomic sequences (≥1 kb) were taxonomically annotated using CAT against the NCBI-nr database (see Footnote 1) with the default parameters (von Meijenfeldt et al., 2019). Sequences classified as belonging to *Synechococcus* (scaffolds ≥1 kb) were retained and deduplicated for the following analysis.

### Community composition and phylogeny of *Synechococcus* based on its sequences

Raw sequencing data from PacBio were processed using Cutadapt (v1.9.1) (Martin, 2011) to retain high-quality sequences between 1.3 and 1.6 kb in length. Then, the sequences were clustered into operational taxonomic units (OTUs) with Vsearch (v1.9.6) (Rognes et al., 2016) and QIIME (v1.9.1) (Caporaso et al., 2010) at a cutoff of 97% identity. The ITS sequences obtained from previous studies were utilized to classify the representative sequences of OTUs. Taxonomy was assigned to the OTUs using the Bayesian algorithm of RDP Classifier. Additionally, a BLASTN search was conducted to assess the similarity between the representative sequences and reference sequences.

**Figure 1:**
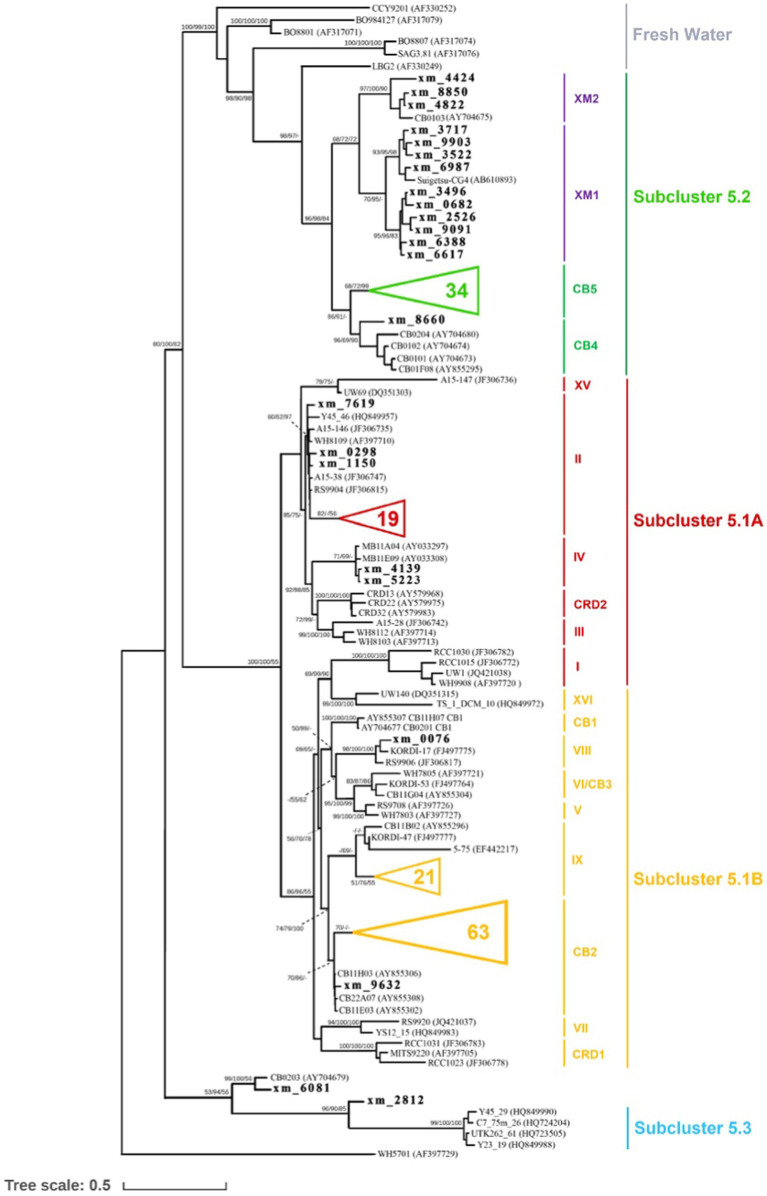
Phylogenetic tree of *Synechococcus*, based on the ITS sequence alignment of 1,388 positions, including tRNAs, showing the phylogenetic relationships among *Synechococcus* genotypes. The two clades identified in this study are depicted in purple. The unclassified representative sequences from our study are indicated in boldface, while some sequences obtained in this study are not shown (represented by triangles). The numbers at the nodes of the tree represent the bootstrap values for maximum likelihood (ML) analyses, posterior probabilities for Bayesian inference (BI), and bootstrap values for the neighbor-joining (NJ) method, respectively.

For the construction of the phylogenetic tree, OTUs that contained a minimum of 10 sequences were chosen. Among these OTUs, representative sequences that exhibited less than 97% similarity to reference sequences were defined as unclassified sequences. A total of 163 unclassified representative sequences were selected and aligned with reference sequences using the MAFFT (v7.508) L-INS-I algorithm (Katoh and Standley, 2013). The alignments were then manually corrected using MEGA X (Kumar et al., 2018; Stecher et al., 2020). After alignment and manual correction, a total of 1,388 positions, including tRNAs, remained. To infer the maximum likelihood (ML) trees, RAxML-NG (v1.1) (Kozlov et al., 2019) was utilized, employing a heuristic search strategy. The ML trees were subjected to a bootstrap test with 1,000 replicates. Bayesian inference was performed using MrBayes (v3.2.7) (Huelsenbeck and Ronquist, 2001; Altekar et al., 2004). The analysis involved running two million generations with 2 Markov chains, and the standard deviation of split frequencies was maintained below 0.05. Additionally, the neighbor-joining (NJ) method was employed for distance analysis in PHYLIP (v3.697) (Felsenstein, 1989). *Synechococcus* sp. WH 5701 was employed as the outgroup for all three phylogenetic tree construction methods described earlier. The ML analysis and Bayesian inference yielded nearly identical tree topologies, while slight variations were observed in the NJ tree (refer to Figure 1).

### Identification of prophages in *Synechococcus* genome sequences

CheckV v0.7.0 (end_to_end) was used to predict prophages that were integrated into host genomes based on the ratio of virus-specific to microbe-specific genes (Nayfach et al., 2020). *Synechococcus* genome sequences with hits to primarily viral databases [VOGDB,^3^ IMG/VR (Paez-Espino et al., 2019), RVDB (Goodacre et al., 2018)] or mapping to viral feature genes (e.g., capsid, terminase) from other databases [KEGG Orthology (Kanehisa and Goto, 2000), Pfam A (El-Gebali et al., 2019), Pfam B (Finn et al., 2010), and TIGRFAM (Haft et al., 2013)] were considered to be virus-specific region-containing sequences. Flanking host regions were removed, and prophage genomes were exclusively retained.

### Identification of viral defense-related genes in *Synechococcus* genome sequences

Antiviral defense-related genes of *Synechococcus* were identified with BLASTp (more sensitive mode, identity ≥30%, E-value <10^−10^) in the DIAMOND program (Buchfink et al., 2015) by searching against the PADS Arsenal database (Zhang et al., 2020). Genes with hits to the PADS database were then imported into HMMScan in the HMMER 3.3 tool suite (Mistry et al., 2013) against PFAM 32.0 (El-Gebali et al., 2019) (E-value <10^−3^, bit score ≥ 30) to check that the identified genes contain conserved domains that have been demonstrated to be involved in prokaryotic defense against phage attack (Doron et al., 2018). Sequences containing conserved domains were retained. The completeness of a defense system was predicted according to the presence of gene components of the system in a sequence as described previously (Dy et al., 2014; Shmakov et al., 2017; Bernheim and Sorek, 2019; Kamruzzaman and Iredell, 2019).

## Results and discussion

### Community composition and phylogeny of *Synechococcus*

**Figure 2:**
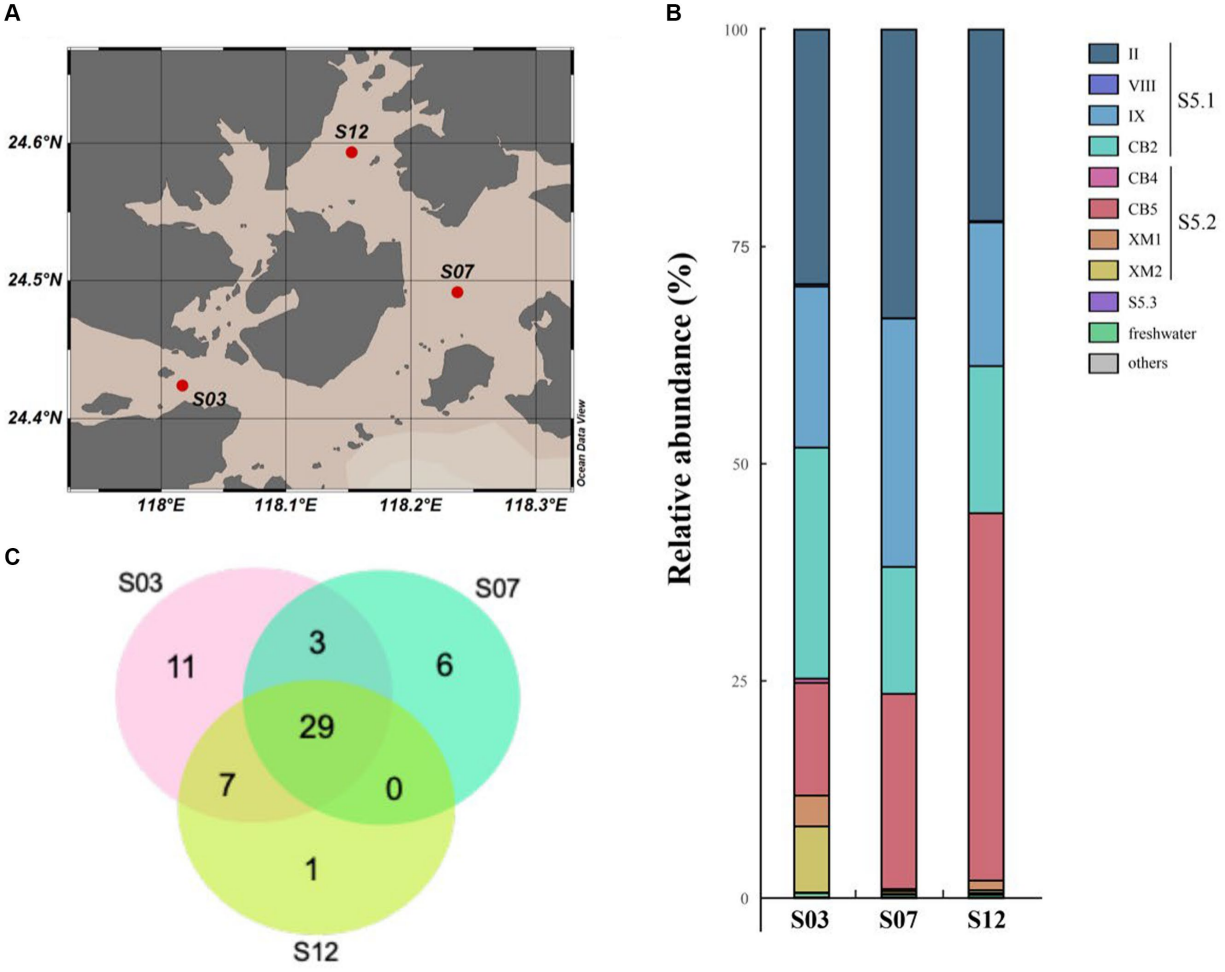
Distribution of the species of *Synechococcus* in Xiamen coastal waters. Map of the sampling station **(A)**. Venn diagram of the species number of *Synechococcus* **(B)**. Community composition of *Synechococcus* in Xiamen coastal waters **(C)**.

Through high-throughput sequencing, we identified a total of 18 clades belonging to three marine *Synechococcus* subclusters [Subclusters 5.1, 5.2, 5.3 (Scanlan et al., 2009)] and three freshwater clusters in our study. In particular, Subclusters 5.1 and 5.2 (referred to as S5.1 and S5.2, respectively) were found to dominate in the coastal waters of Xiamen (Figure 2). S5.1 encompassed 16,371 OTUs (85,376 sequences), while S5.2 included 9,875 OTUs (37,727 sequences). In contrast, Subcluster 5.3 and freshwater *Synechococcus* had lower representation, with only 48 OTUs (137 sequences) and 74 OTUs (388 sequences), respectively. Our analysis of geographical position, salinity, and nitrogen data from the three stations (S03, S07, and S12) suggested that the environmental conditions at each station were distinct (Supplementary Figures S1, S2). Station S03, affected by the input of freshwater from the Jiulong River, is a typical brackish water region that exhibits lower salinity and reduced water transparency (Maskaoui et al., 2002; Xue et al., 2004). At this station, *Synechococcus* shows high abundance and genetic diversity, and two novel clades, XM1 and XM2, were identified, which were not fully recognized previously. On the other hand, station S07, located close to the offshore environment, has oceanic-like environmental conditions and harbors a greater presence (25,724 sequences of 7,448 OTUs) of Subcluster 5.1. Station S12 is located within the semienclosed Tong’an Bay, where a total of 14,055 sequences of 3,961 OTUs were assigned to S5.2, indicating both high species diversity and high abundance of S5.2. These findings suggest that the *Synechococcus* community is sensitive to environmental changes in the coastal water of Xiamen Island.

In our study, over half of the sequences were classified into S5.1, with a major presence in clades II, IX, and CB2. Clade II, known for its wide ecological distribution across the global ocean, emerged as the dominant group of marine *Synechococcus* (Fuller et al., 2003; Zwirglmaier et al., 2008; Ahlgren and Rocap, 2012). Clade CB2, initially identified in Chesapeake Bay in 2006, exhibited a widespread distribution in the East China Sea (Chen et al., 2006; Choi and Noh, 2009). Notably, our study area showed a high abundance of clade IX, indicating a potentially greater prevalence in estuarine waters than previously anticipated, consistent with the findings of earlier investigations (Fuller et al., 2003; Zwirglmaier et al., 2008; Ahlgren and Rocap, 2012).

Based on previous studies (Cai et al., 2010; Ahlgren and Rocap, 2012), *Synechococcus* S5.2 is primarily composed of two clades, namely CB4 and CB5. These clades have been found to dominate in freshwater, estuarine, and brackish waters (Chen et al., 2004). Their dominance in marine habitats may be attributed to unique salinity adaptation mechanisms and pigment types, specifically PC-rich pigments (Xia et al., 2023). However, in our study, we identified 851 and 1,042 OTUs within S5.2, forming two distinct lineages (Figure 1) named clades XM1 and XM2, which have not been previously defined. A reference strain of *Synechococcus* (Suigetsu-CG4) belonging to clade XM1 was isolated from the hypoxic boundary zone of Lake Suigetsu in Japan (35°35’N, 135°52′E) (Ohki et al., 2012). This strain is characterized by the absence of phycoerythrobilin and a preference for growth in environments with low light and low salinity. A cultured strain of *Synechococcus* CB0103, which was previously isolated from Chesapeake Bay (Chen et al., 2006), clustered together with at least three OTUs identified in our study. These sequences formed the clade XM2, which exhibited dominance in S03. Building upon previous studies on CB0103 (Murrell and Lores, 2004), *Synechococcus* communities appear to be highly responsive to environmental changes including the influence of freshwater input and fluctuations in salinity, nutrient levels, and other environmental factors. Their influence may significantly impact the ecological distribution of *Synechococcus*. Furthermore, our findings suggest that some potential clades within S5.2 may not be fully characterized, and the complex habitat implies that S5.2 is more genetically diverse.

### Metabolic pathways of *Synechococcus*

**Figure 3:**
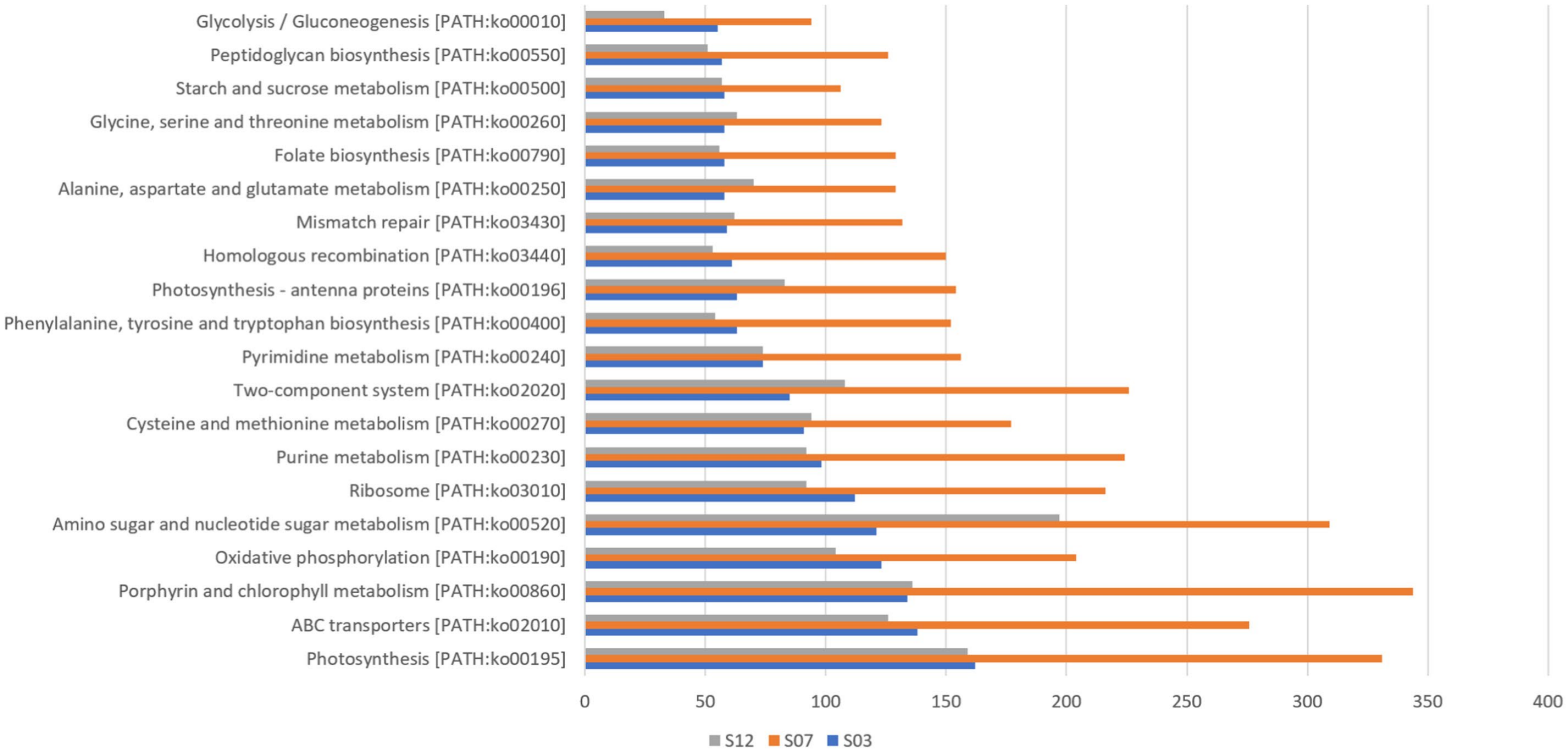
Top 20 metabolic pathways of *Synechococcus.*

In this study, we detected 101 *Synechococcus* species with genomic taxonomic annotation information (Supplementary Table S1) and annotated a total of 226 different functional genes using the KEGG database (Figure 3). Among these genes, those related to photosynthesis [PATH: KO00195] had the highest count at all three sites. Additionally, we observed that genes related to ABC transport, porphyrin, and chlorophyll metabolism also had relatively high gene numbers across all three sites. These results suggest that photosynthesis, substance synthesis, and transport are likely to be key biological processes in *Synechococcus* and provide a potential molecular basis for understanding their photosynthetic capability.

By comparing genomic data with 343 known *Synechococcus* genomes, several unmatched ORF sequences, were discovered (Supplementary Tables S2, S3). These unmatched sequences are likely to be unique coding genes of *Synechococcus* in the Xiamen region and may be linked to its adaptability to the environment. For instance, the most abundant genes related to amino sugar and nucleotide sugar metabolism aid in the synthesis of sugar complexes required for cell walls, providing more protection to *Synechococcus* in complex coastal waters.

Additionally, we detected genes related to photosynthesis in the *Synechococcus* strain, with a total of 284 genes present in five genome bins. Some of these genes are shown in Supplementary Figure S3. We also detected 12 phycobilisome-related genes in the five *Synechococcus* genomes, derived from four classes of genes: APCF (phycobilisome core component), APCC (phycobilisome core connexin), cPCG (core-linked protein), and APCE (core-membrane-linked protein). Notably, no *cpcD* gene was found in these *Synechococcus* species, which may indicate that the *cpcD* gene was deleted during evolution. This is consistent with previous reports that *cpcD* is absent in many *Synechococcus* strains, such as *Synechococcus* RCC307 and *Synechococcus* CC9902 (Six et al., 2007). We found that photosystem II is associated with more Psb-coding genes, including *psbU* and *psbV*. However, *Prochlorococcus*, a close relative of *Synechococcus*, has evolved to have smaller genomes, resulting in the lack of genes encoding photosystem II foreign proteins, including PsbU, PsbV, and PsbQ. *psbU*, *psbV*, and *psbQ* encode proteins associated with the oxidative complex of photosystem II, and their absence may affect the stability of photosystem II and increase its sensitivity to various stresses. Nevertheless, we found that *Synechococcus* possess numerous related genes and lack only the *psbQ* gene, which may make them less sensitive to light stress.

We also detected a total of 348 genes associated with carbohydrate metabolism, which are involved in 15 different carbon metabolic pathways. Interestingly, we found that almost all the genes required for core carbon metabolic pathways were present in M-S12-8_bin36, providing the potential for efficient carbohydrate metabolism in *Synechococcus* in Xiamen, as illustrated in Supplementary Figure S4. Furthermore, we discovered the presence of a nitrogen metabolism gene, *ntcA*, in *Synechococcus*. This gene was detected in M8-S12, M8-S07, and M8-S03, suggesting its potential significance in marine *Synechococcus* in Xiamen. These findings suggest that *Synechococcus* can efficiently absorb carbon and nitrogen from the environment for the synthesis of other compounds, which may contribute to their adaptation to the local environment.

### Benefits from metabolic genes in prophages

**Figure 4:**
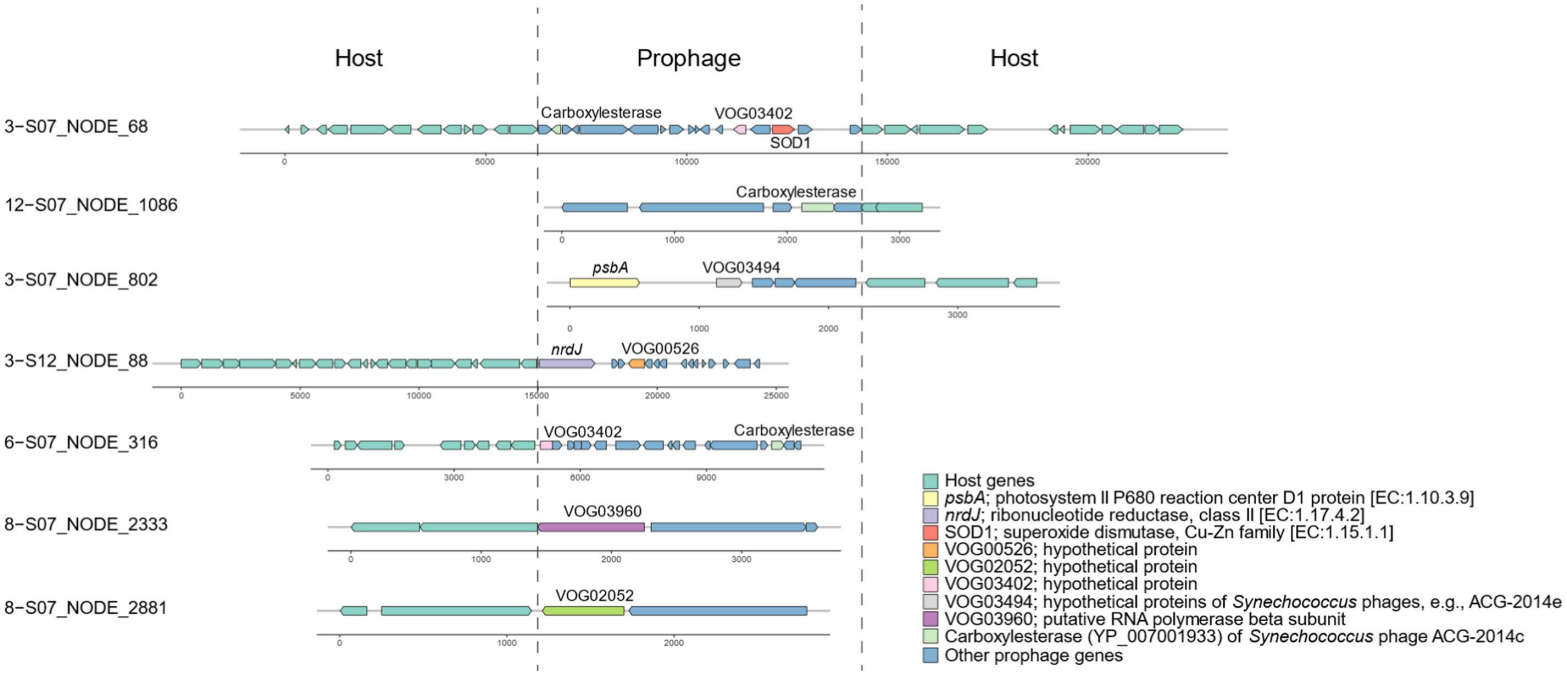
Representatives of putative prophages in *Synechococcus* genomes.

Based on the identification of phage-specific and host-specific genes, we detected 89 incomplete prophage regions in the *Synechococcus* genomes (Supplementary Table S4). All these prophage fragments contained at least one viral gene with hits against viral databases; for example, VOG03402 was located on scaffold 3-S07_NODE_68 (Figure 4). According to functional annotation, 23 prophage genes were likely to be involved in host metabolism of various substances, including carbohydrates, energy, nucleotides, and amino acids (Supplementary Table S5). Specifically, these metabolic phage genes were related to nine pathways, namely, photosynthesis, glycolysis/gluconeogenesis, purine metabolism, cysteine and methionine metabolism, phenylalanine, tyrosine and tryptophan biosynthesis, aminoacyl-tRNA biosynthesis, ABC transporters, homologous recombination, and peroxisomes. For example, for photosynthesis, prophage-encoded *psbA* for the photosystem II P680 reaction center D1 protein was detected near VOG03494 of *Synechococcus* phages on scaffold 3-S07_NODE_802 (Figure 4). In the metabolism of nucleotides, the ribonucleotide reductase *nrdJ* was identified in 3-S12_NODE_88, which contained the viral gene VOG00526 (Figure 4).

The identification of incomplete prophages in *Synechococcus* bacteria of Xiamen seawater is consistent with the evidence of phage remnants in the genomes of marine unicellular *Synechococcus* isolates (Flores-Uribe et al., 2019). The incomplete prophages might be trapped phage elements resulting from host genome recombination in their evolutionary history (Canchaya et al., 2003) and finally lost their ability to switch to a lytic cycle and become cryptic prophages (Wang et al., 2010). However, cryptic prophages can express their genes and benefit their hosts, for example, in terms of stress resistance, which demonstrates the function of prophages, even though they are not intact (Wang et al., 2010). According to the evidence, genes in the predicted prophages in *Synechococcus* of Xiamen seawater are proposed to be functional and can contribute to host metabolism. For instance, given the expression of prophage-encoded *psbA* on scaffold 3-S07_NODE_802 of *Synechococcus*, prophages can participate in the photosystem II P680 reaction to facilitate photosynthesis. Genes related to many other pathways, such as cysteine and methionine metabolism, can allow prophages to play a role in nutrient biosynthesis to enhance host fitness. Additionally, *Synechococcus* prophages carry genes for general L-amino acid transport system permease proteins that are components of ABC transporter systems, indicating that prophages also play a role in the transport of synthesized organic matter, such as amino acids, and metals. The presence of copper-zinc superoxide dismutase (SOD1 protein) aligns with the prior finding that superoxide dismutase exists in a coastal cyanobacterium, *Synechococcus* sp. strain CC9311 (Palenik et al., 2006). This implies that prophages may play a role in metal metabolism, enabling them to detect and react to variations in the coastal environment. Overall, various genes carried by prophages can contribute to *Synechococcus* hosts in terms of fitness to accommodate the coastal marine environment.

### Defense-related genes against phage attack

**Figure 5:**
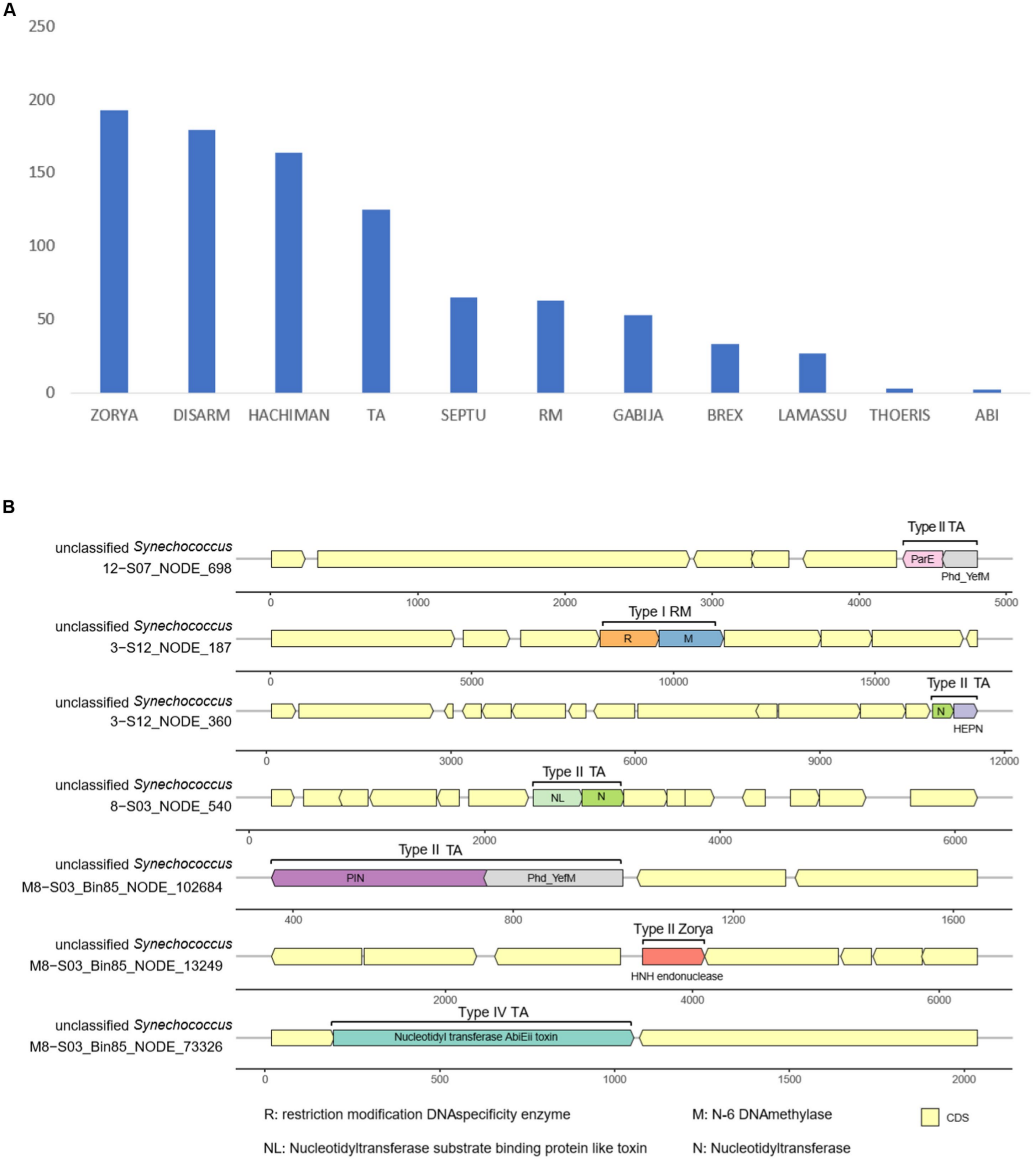
Count of antiviral defense-related genes in *Synechococcus* genomes **(A)** and antiviral defense systems in *Synechococcus* genomes **(B)**.

A total of 773 genes harbored in the 16,258 *Synechococcus* sequences were detected to be associated with diverse antiviral defense systems (Supplementary Table S6). These systems include Zorya, DISARM, Hachiman, toxin–antitoxin (TA), Septu, RM, Gabija, Brex, Lamassu, Thoeris, and abortive infection (Figure 5A). Among these systems, Zorya, DISARM, Hachiman, and TA accounted for a large proportion of genes (more than 450 genes). Additionally, the systems of Zorya and TA, along with the less abundant RM, were found to be complete. The complete system types with all the required gene components included Type II Zorya, Type II/IV TA, and Type I RM (Figure 5B). Type II Zorya was composed of ZorE genes for HNH endonucleases (Pfam accession: PF01844), for example, on scaffold M8 − S03_Bin85_NODE_13249 of *Synechococcus* bin M8 − S03_Bin85. The Type II TA was constituted by multiple toxin–antitoxin gene pairs such as genes for ParE toxin and Phd_YefM antitoxin on the scaffold 12 − S07_NODE_698, HEPN and nucleotidyltransferase domains on 3 − S12_NODE_360, nucleotidyltransferase substrate binding protein-like toxin and nucleotidyltransferase on 8 − S03_NODE_540, and PIN and Phd_YefM on M8 − S03_Bin85_NODE_102684 (Figure 5B). Type IV TA is a system consisting of the nucleotidyl transferase AbiEii toxin gene, as evidenced by the genome structure of the scaffold M8 − S03_Bin85_NODE_73326. The Type I RM, which is a foreign DNA-targeting RM system, encodes a gene pair, for example, located on 3 − S12_NODE_187, for a methyltransferase for methylation and a restriction enzyme for DNA cleavage.

Targeting invading DNA, reducing cell activity, and conducting programmed cell death are common ways to prevent phage infection and avoid population decimation (Rostøl and Marraffini, 2019). To target external DNA, bacteria can depend on innate immunity, including the systems of RM (Ershova et al., 2015) and DISARM (Ofir et al., 2018). Bacteria can also use the TA (Yamaguchi et al., 2011) and abortive infection (Chopin et al., 2005) systems to reduce cell activity, thereby preventing phage replication. Additionally, bacteria may depend on more systems, for example, Zorya, Hachiman, Gabija, Septu, Thoeris, and Lamassu, to fight invading phages (Doron et al., 2018). In seawater, cyanophages are plentiful and can infect and lyse approximately 20–40% of cyanobacteria in the ocean daily (Proctor and Fuhrman, 1990). Moreover, phages isolated from Xiamen seawater demonstrate a robust lytic capability with a significant burst size (Ma et al., 2021). This evidence highlights the substantial threat posed by viruses to the *Synechococcus* community. However, our metagenomic exploration showed that *Synechococcus* of Xiamen seawater contained hundreds of genes related to 11 defense systems against phage infection. The presence of multiple systems suggests that *Synechococcus* in Xiamen seawater may have evolved multiple complementary defense lines (Dupuis et al., 2013), which can enhance the bacterial ability to prevent phage DNA injection and replication (Rostøl and Marraffini, 2019). For example, when cyanophages inject their DNA into *Synechoccocus* cells, RM systems such as Type I RM located on the scaffold 3 − S12_NODE_187 (Figure 5B) can activate the expression of genes encoding restriction endonucleases to cleave injected DNA. Simultaneously, TA systems such as Type II TA located on scaffold 3 − S12_NODE_360 (Figure 5B) can induce the dormancy of *Synechococcus* cells to inhibit the expression of invaded phage genes. For other systems newly discovered with unknown mechanisms, for instance, Zorya and Hachiman, we assume that they have different functional roles in the prevention of phage infection. Overall, the presence of abundant and various defense systems suggests a potential deployment of multiple defense lines and reflects the adaptation of *Synechococcus* to the environment of Xiamen seawater where heterogeneous phage predators may co-occur.

## Conclusion

In this study, we identified a diverse array of *Synechococcus* clades inhabiting the coastal waters of Xiamen. These clades are categorized into three marine subclusters and three freshwater clusters. Through our analysis, we unveiled a rich set of *Synechococcus* species, comprising over 25,000 OTUs. Our taxonomic annotations provided insights into the presence of more than 100 distinct *Synechococcus* species. Furthermore, we delved into the functional annotation of their genomes, uncovering a multitude of functional genes associated with various pathways. These pathways encompass critical processes such as photosynthesis, ABC transport, porphyrin metabolism, and chlorophyll metabolism. In the exploration of these genomes, we identified specific genes associated with phages and host interactions, which led to the discovery of incomplete prophage regions integrated into the *Synechococcus* genomes. These prophage regions harbored metabolic genes involved in diverse processes, including carbohydrate metabolism, energy production, nucleotide synthesis, metal transport, and amino acid metabolism. This implies that prophages participate in a wide range of host metabolic activities, enhancing the adaptability of their *Synechococcus* hosts to the ever-changing coastal marine environment. Moreover, our analysis revealed a substantial number of genes linked to antiviral defense systems within the *Synechococcus* genomes. These defense systems encompass a range of mechanisms, including innate and adaptive immunity systems, such as the RM system. Additionally, we identified several previously unknown defense mechanisms, such as Gabija, Brex, Lamassu, and Thoeris, which may play a crucial role in bolstering the defense capabilities against viral threats. The presence of these abundant and diverse defense systems indicates the deployment of multiple layers of defense strategies, underscoring the adaptability of *Synechococcus* to the dynamic environment of Xiamen seawater, where a heterogeneous community of phage predators may be present.

## Data availability statement

The sequencing raw data that support the findings of this study are deposited in the NCBI database under the BioProject ID PRJNA1030774.

## Author contributions

TZ: Conceptualization, Data curation, Formal analysis, Investigation, Methodology, Visualization, Writing – original draft. KZ: Conceptualization, Data curation, Formal analysis, Investigation, Methodology, Visualization, Writing – original draft. YW: Formal analysis, Visualization, Writing – original draft. JX: Formal analysis, Visualization, Writing – original draft. QZ: Supervision, Writing – review & editing. TL: Funding acquisition, Project administration, Supervision, Validation, Writing – review & editing. NJ: Funding acquisition, Project administration, Supervision, Writing – review & editing.

## Footnotes

^1^ http://blast.ncbi.nlm.nih.gov/database

^2^ http://merenlab.org/software/#anvio

^3^ http://vogdb.org
