# Supplementary material for: Genomic insights into the adaptation of Synechococcus to the coastal environment on Xiamen
Source: Front Microbiol. 2023 Nov 20;14:1292150. doi: 10.3389/fmicb.2023.1292150 (PMC10696648; doi:10.3389/fmicb.2023.1292150)
Supplement: Supplementary file 2 [file Data_Sheet_1.docx]

**Supplementary Methods**

**Identification of genes that are likely specific to Xiamen *Synechococcus***

The genome sequences of *Synechococcus* identified in this study were imported into PRODIGAL (parameters:-m, -p meta) to predict the Open Reading Frames (ORFs) (Hyatt *et al.*, 2010). The *Synechococcus* ORFs predicted in the study were aligned with 343 known *Synechococcus* genomes downloaded from NCBI GenBank (accessed on 14 February 2021; query title: search *Synechococcus*) using BLASTn (E-value <10). The unmatched ORFs of *Synechococcus* in our study were defined as a gene of Xiamen *Synechococcus* in the coastal waters of. The functional annotation of the ORFs was then conducted by searching against KEGG database using KofamScan version 1.2.0 (E-value <0.00001) (Aramaki *et al.*, 2020).

**References**

Aramaki, T., Blanc-Mathieu, R., Endo, H., Ohkubo, K., Kanehisa, M., Goto, S., and Ogata, H. (2020) KofamKOALA: KEGG Ortholog assignment based on profile HMM and adaptive score threshold. *Bioinformatics* **36**: 2251-2252.

Hyatt, D., Chen, G.L., Locascio, P.F., Land, M.L., Larimer, F.W., and Hauser, L.J. (2010) Prodigal: prokaryotic gene recognition and translation initiation site identification. *BMC Bioinf* **11**: 1-11.

**Supplementary Figures**

**Fig. S1 Line chart of seawater salinity of three stations (S03, S07, and S12) in Xiamen Island**. The vertical axis represents salinity (‰). The horizontal axis represents sampling date (year/month/day). At each station, salinity was measured using YSI measuring instrument.

**Fig. S2 Line chart of nitrite concentration in seawater of three stations (S03, S07, and S12) in Xiamen Island.** The vertical axis represents nitrite NO_3_ (μmol/L). The horizontal axis represents sampling date (year/month/day). Nitrogen concentrations (μmol/L) was measured with PowerMon Kolorimeter AA3 Automatic nutrient analyzer ((Bran+Luebbe, Charlotte, NC, USA)) to evaluate the effects of nutrients on the community structure of *Synechoccocus*.

**A. Photosynthesis-related genes of the *Synechococcus* M-S07-8_bin1**


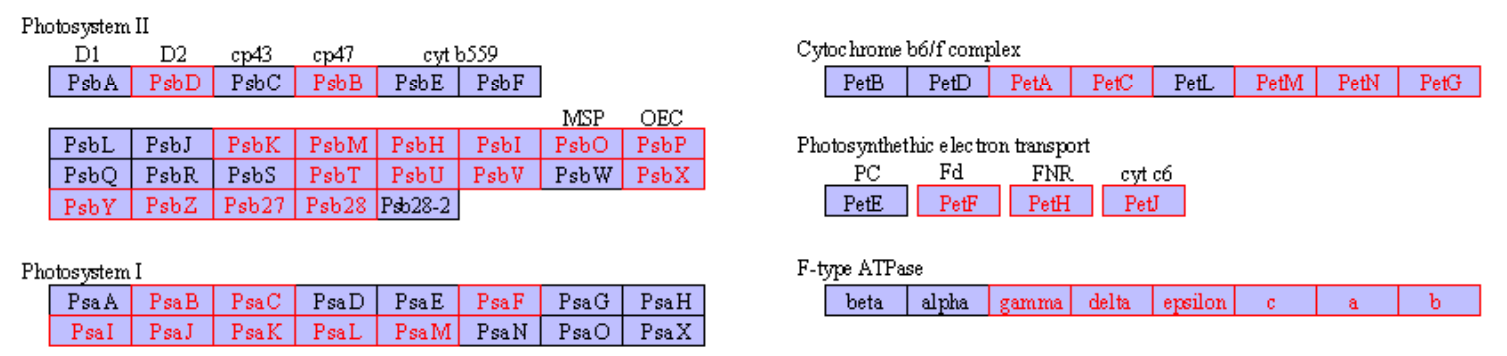


**B. Photosynthesis-related genes of the *Synechococcus* M-S07-8_bin5**


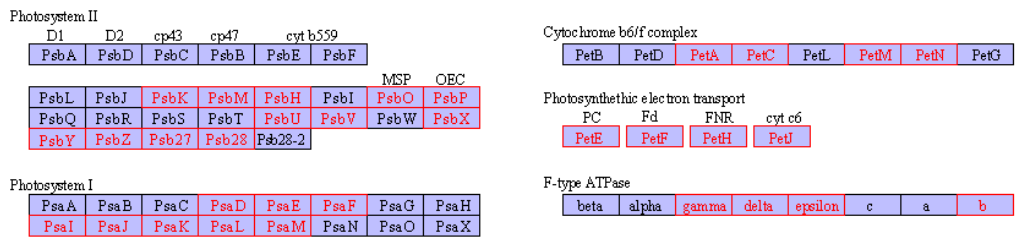


**C. Photosynthesis related genes of the *Synechococcus* M-S03-8_bin30**


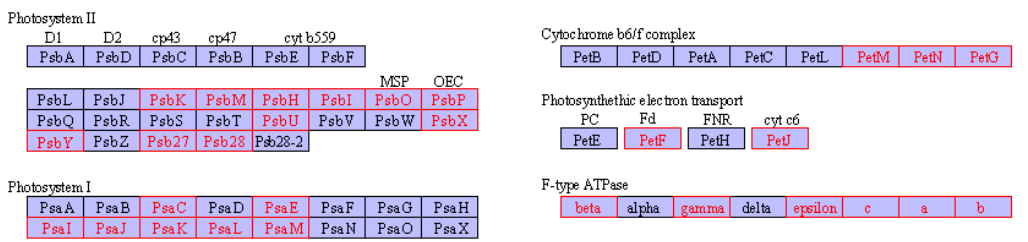


**D. Photosynthesis related genes of the *Synechococcus* M-S12-8_bin36**


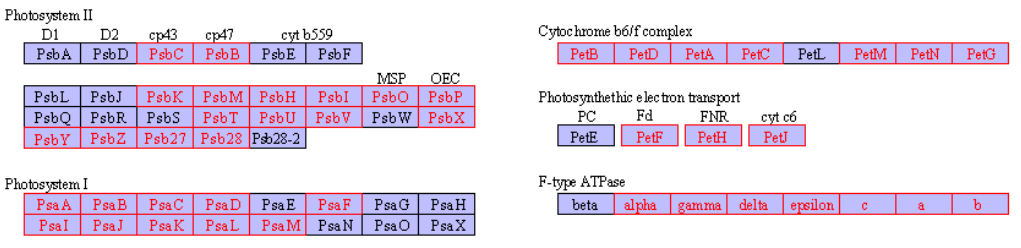


**E. Photosynthesis related genes of the *Synechococcus* M-S03-8_bin85**


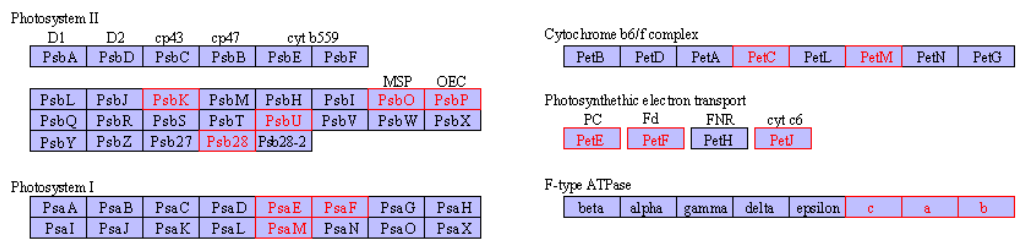


**Fig. S3 A plot of photosynthesis systems and photosynthesis related genes in Xiamen *Synchococcus* genome bins.** Photosynthesis related genes present in *Synchococcus* genomes in the study were marked in red.

**A.**


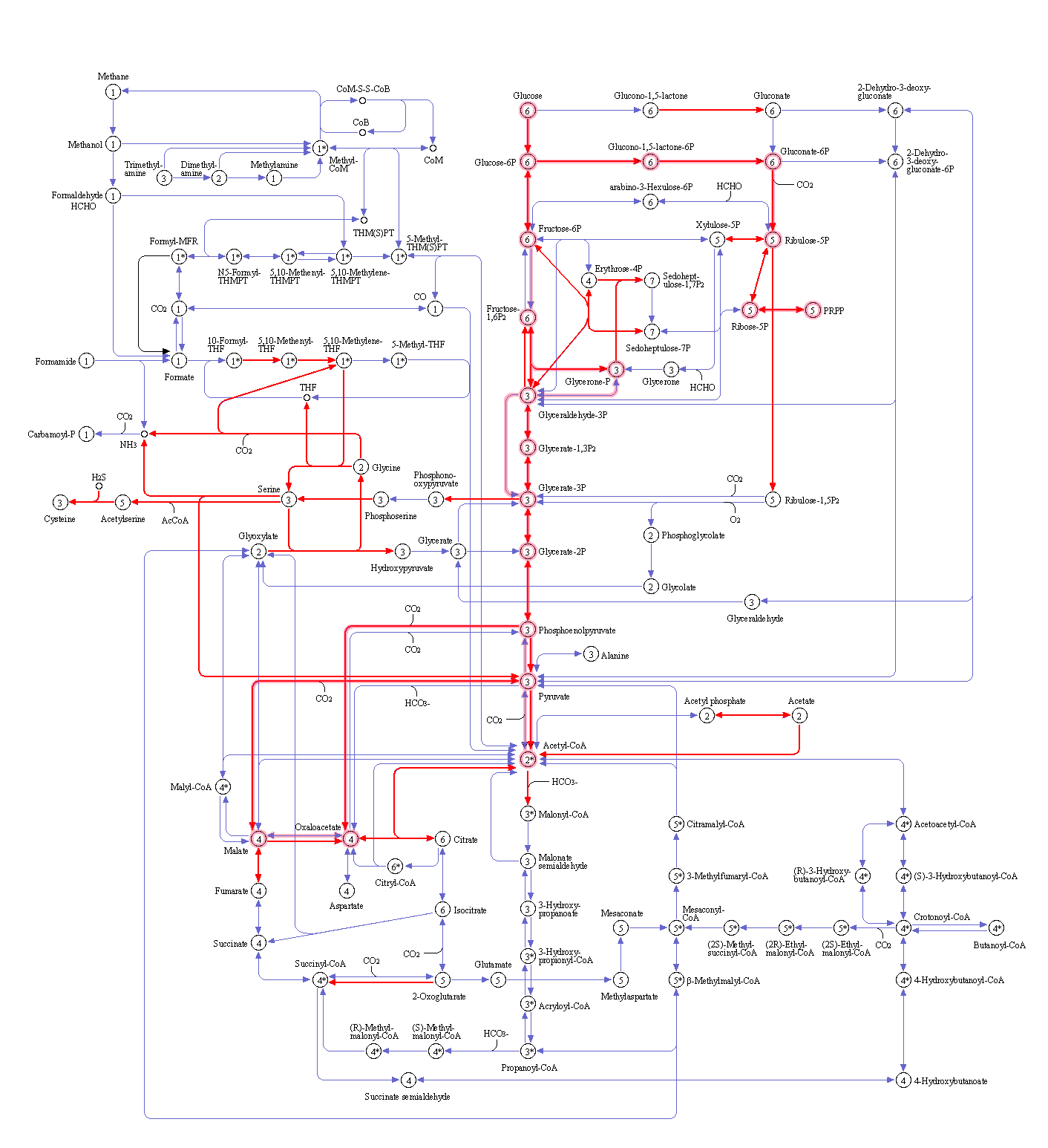


**B. C.**


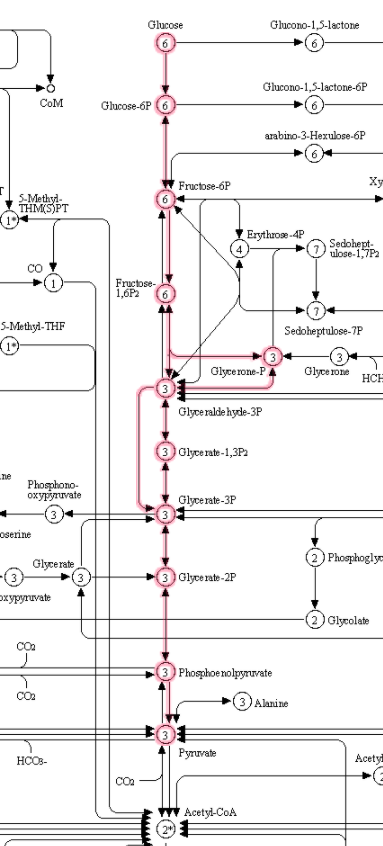

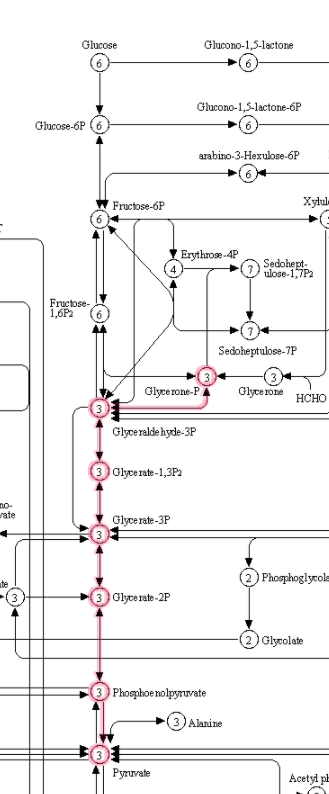


**D. E.**


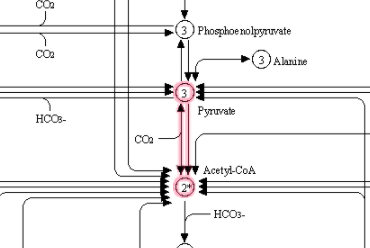

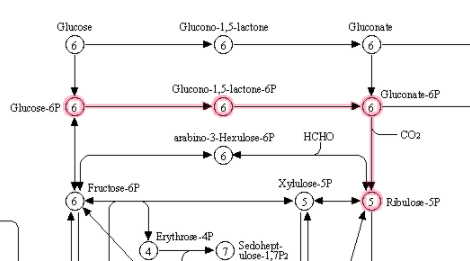


**F. G.**


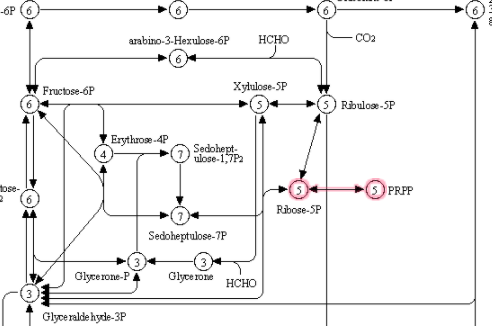

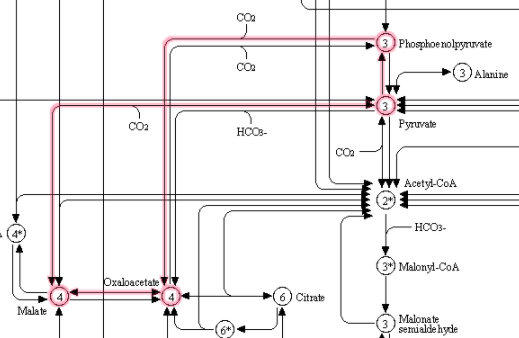


**Fig. S4 Carbon metabolism pathways in the Xiamen *Synchococcus* genome bin M-S12-8_bin36.** Derived from kegg annotation results, the red font indicates that the gene has been detected, and the pink marker indicates that the metabolic pathway has a high degree of completeness. M-S12-8_bin36 is involved in a number of core carbon metabolism-related pathways (A), including the Embden-Meyerhof pathway glycolysis (B), which involves three-carbon compound glycolysis (C), pyruvate oxidation (D), oxidative phase pentose phosphate pathway (E), PRPP biosynthesis (F), CAM sedum metabolism (G).
